# A_2A_R Antagonism with DZD2269 Augments Antitumor Efficacy of Irradiation in Murine Model

**DOI:** 10.7150/jca.43966

**Published:** 2020-03-26

**Authors:** Jiaqi Huang, Di Zhang, Yu Bai, Pamela Yang, Ligang Xing, Jinming Yu

**Affiliations:** 1 Department of Clinical Medicine, Shandong University, Jinan, Shandong 250000, P.R. China.; 2 Department of Radiation Oncology, Shandong Cancer Hospital and Institute, Shandong First Medical University and Shandong Academy of Medical Sciences, Jinan, Shandong 250117, P.R. China.; 3 Dizal (Jiangsu) Pharmaceutical Co., Ltd., Wuxi, Jiangsu 214028, P.R. China.

**Keywords:** adenosine, A_2A_R, irradiation, immunotherapy

## Abstract

Accumulated extracellular adenosine suppresses antitumor immunity via adenosine 2A receptor (A_2A_R). Blockade of A_2A_R with DZD2269 can inhibit phosphorylation of cAMP response element-binding protein mediated by adenosine analogue *in vitro* and *in vivo.* Irradiation can cause the release of adenosine and lead to a rapid increase in free extracellular adenosine in the tumour area. DZD2269, a novel A_2A_R Antagonism, induces incomplete antitumor responses in multiple syngeneic mouse tumour models. Combining DZD2269 with IR can induce a synergistic anticancer effect. IR increases the infiltration of various subtypes of T cells, including CD4+, CD8+ and Foxp3+ T cells, into the tumour area. Combining IR and DZD2269 improves the tumour immune microenvironment, leading to suppressed infiltration of regulatory T (Treg) cells and enhanced IFN-γ expression by tumour-infiltrating lymphocytes. The results support the use of A_2A_R antagonism with DZD2269 as a therapeutic strategy for monotherapy or combination therapy with IR.

## Introduction

Immunosuppression plays a key role in cancer progression and metastasis. Recently, immunotherapy has produced dramatic antitumor efficacy in a subset of patients. However, only a fraction of patients benefit from immunotherapy, due to some factors within the tumor microenvironment (TME) that mitigate immune cell infiltration and suppress antitumor immune responses 1. A group of surface receptors on T cells, called immune checkpoint proteins, deliver negative signals to T cells upon engagement of the corresponding ligand expressed on antigen-presenting cells 2. Tumor cells can exploit the inhibitory mechanism by expressing the immunosuppressive ligands, and preventing the binding of immune checkpoint molecules, such as PD-1 and CTLA-4, by monoclonal antibodies has been found to overcome immunosuppression in cancer therapy^3^,^4^.

Increasing evidence suggests that accumulated extracellular adenosine in the TME can promote tumour growth, suppress antitumor immunity and limit the efficacy of antitumor therapy 5, 6. The generation of adenosine was correlated with the resistance to chemotherapy and poor survival in triple-negative breast cancer patients 7. Furthermore, increased extracellular adenosine levels can protect tumour cells from irradiation (IR)-induced damage and impair the antitumor response 8. The adenosine 2A receptor (A_2A_R) pathway is the main pathway through which adenosine produces immunosuppressive effect 9, 10. Stimulation of A_2A_R induces the phosphorylation of cAMP response element-binding protein (CREB) 11. The inhibition of phosphorylated CREB (pCREB) production induced by stimulation with the high-affinity, stable adenosine analogue NECA is frequently used as a readout for the activity of A_2A_R antagonists 12. In preclinical models A_2A_R antagonism showed antitumor effects 13,14. Therefore, combining A_2A_R antagonism with IR may enhance the curative effect of radiotherapy.

In this study we reported cooperative antitumor response and explored underlying mechanism of the combination of a novel A_2A_R antagonist DZD2269 and IR in murine cancer model. Our results provided support for combination treatment with A_2A_R antagonism and radiotherapy as promising therapeutic strategy in future clinical trials.

## Materials and Methods

### Cell lines and reagents

Mouse colon cancer cell line MC38 was obtained from the National Cancer Institutes of Health (NIH). Mouse triple negative breast cancer cell line 4T1 was purchased from the American Type Culture Collection (ATCC). MC38 cells were cultured in DMEM (Gibco, Cat. No. 12430-054) + 10% fetal bovine serum (FBS, Gibco, Cat. No. 10091-148) + 1% MEM NEAA (Gibco, Cat. No. 11140-050) + 1% sodium pyruvate (Gibco, Cat. No. 11360-070). 4T1 cells were cultured in RPMI medium 1640 (Gibco, Cat. No. 22400-089) + 10% FBS (Gibco, Cat. No. 10091-148). For adenosine detection *in vitro*, 10 μM hydrochloride (EHNA; Sigma, Cat. No. 58337-38-5), 10 μM 5-Iodotubericidin (5-ITU; Sigma, Cat. No. 24386-93-4) and 0.5 μM dipyridamole (Sigma, Cat. No. 58-32-2) were added into medium.

DZD2269 was provided by Dizal Pharma (Wuxi, China) and was dissolved in dimethyl sulfoxide for *in vitro* study. For *in vivo* study, DZD2269 was prepared with a final concentration of 0.3 mg/mL in 0.5% hydroxy propyl methyl cellulose (HPMC; Sigma, Cat. No. 9004-67-5) and 0.1% Tween-80 (Sigma, Cat. No P1754).

### Mouse model

Six- to eight-week-old C57BL/6 and Balb/c mice were purchased from Charles River. Mice were fed in the animal facility at Dizal Pharma for one week prior to tumor engraftment. All animal experiments were approved by the Institutional Animal Care and Use Committee (IACUC) of Dizal Pharma. Tumour cells were suspended in cold serum-free medium at a density of 1×10^7^/mL, and 100 μL cell suspension was subcutaneously injected onto the dextral lower-back region of each mouse. Tumour diameter was measured with digital callipers every 2-3 days. Mice were euthanatized when the tumour volume reached 1,500 mm^3^ or with weight loss ≥ 20%.

### Microdialysis

Microdialysis probes were surgically implanted into an established tumour bulk whose largest diameter had reached 10 mm. Microdialysis was performed with a flow rate of 2 μL/minute. PBS supplemented with 0.2 μM CaCl_2_ and 0.1 μM MgCl_2_ was used as perfusion fluid; 10 μM EHNA and 10 μM 5-ITU were added to the perfusion fluid within 2 h before use. Concentrations of adenosine were determined by high-performance liquid chromatography (HPLC) with quadrupole mass spectrometry detection using an ACQ Triple Quad 5500 instrument (Acquity UPLC, USA). An amide 1.7 μm column (Acquity UPLC, USA) was used for detection. Data were calibrated and quantified with Analyst system (Acquity^TM^, version 1.6.1).

### Measurement of CREB phosphorylation

Cell lysates were collected after the cells were lysed in lysis buffer (BD, Cat. No. 558049). The lysates were stained with antibody cocktail containing anti-CD8a FITC (BD, Cat. No. 553031), anti-CD45 PE (BD, Cat. No. 553081), and anti-pCREB AF647 (CST, Cat. No. 14001) for 1 h at room temperature in the dark. Cells were then washed twice with FACS buffer and analyzed on a flow cytometer (Cell Analyzer FACS canto). The mean fluoresce intensity (MFI) of CREB phosphorylation signal was measured.

### Immunohistochemistry (IHC)

Tumor tissues were collected 17 days after tumor engraftment. IHC was performed on 3 μm FFPE sections using a Lab Vision autostainer (Thermo). Then, the slides were subjected to antigen retrieval for 15 min followed by incubation with endogenous peroxidase block for 10 min. The sections were incubated with primary antibodies for CD4 (CST, Cat. No. ab183685), CD8 (CST, Cat. No. CST98941) and Foxp3 (CST, Cat. No. CST12653) for 1 h at room temperature, then with Envision+ System HRP- Labelled Polymer Anti-Rabbit (DAKO, Cat. No. K4003) for 30 min and developed in diaminobenzidine substrate for 5 min. Then, the sections were counterstained, dehydrated and cleared in the Leica XL autostainer. Positive cell percentage of stained IHC slides was quantified with a HALO^TM^ system.

### In situ hybridization (ISH)

Tumor tissues were processed as described for IHC, and then incubated with H_2_0_2_ and Protease Plus reagents (ACDbio, Cat. No. 322330; DAKO, Cat. No. K3468), and placed in 40°C preheated hybridization buffer (ACDbio, Cat. No. 310013) for 30 min. The samples were washed and incubated with 2.5 HD Detection Kit-BROWN (ACDbio, Cat. No. 322310), the slides were then incubated with DAB for 5 min and counterstained. The foci number per stained slides was quantified with a HALO^TM^ system.

### Statistical analysis

Data were shown as the mean ± SEM. Two-way ANOVA was applied for comparison of different treatment groups. A *p* value of <0.05 was considered statistically significant.

## Results

### DZD2269 blocked A_2A_R activation on T cells *in vitro* and *in vivo*

**Figure 1:**
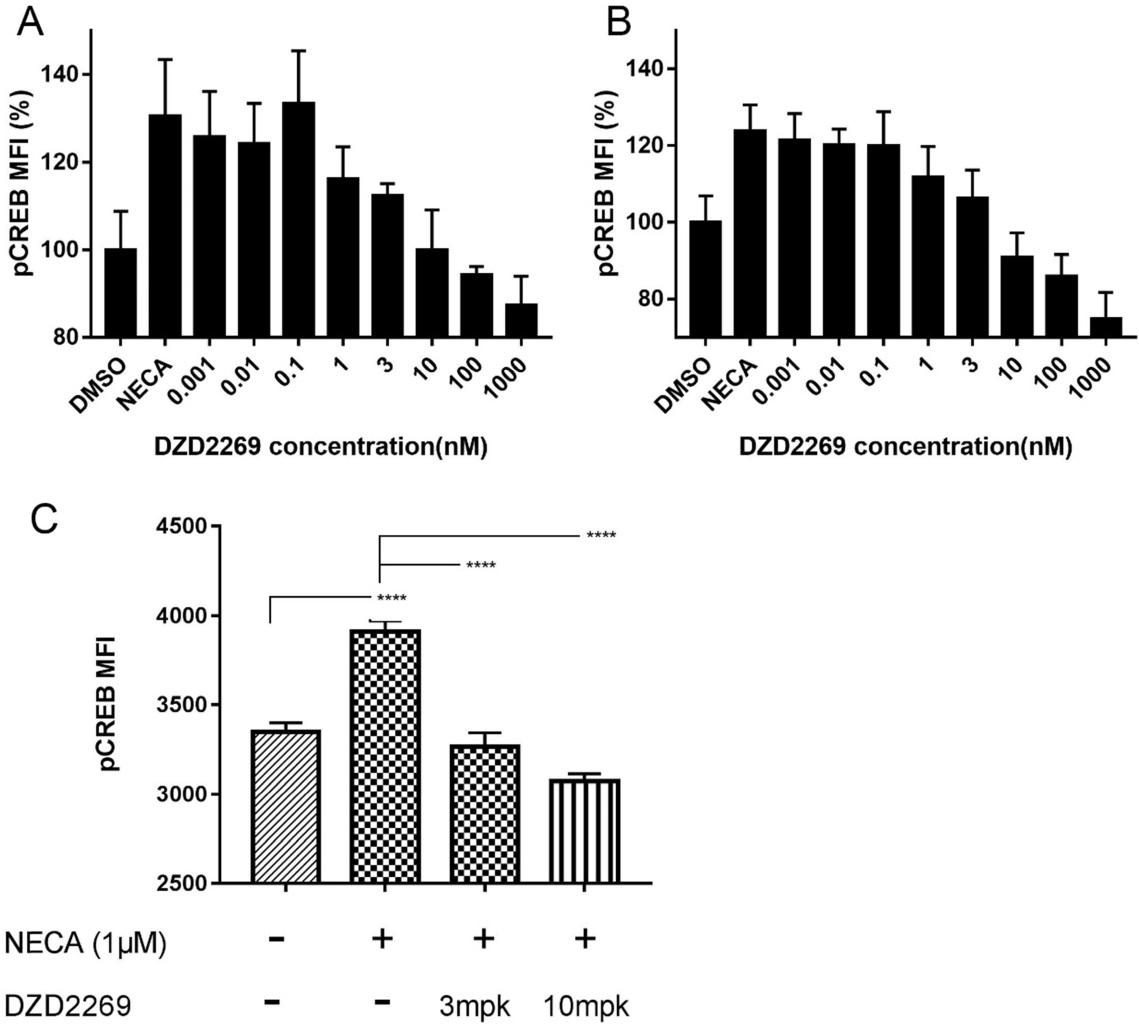
** Blockage of A2AR with DZD2269 inhibited CREB phosphorylation in T cells.** Mean fluorescence intensity (MFI) of CREB phosphorylation signal compared to DMSO control was measured in mouse **(A)** CD8+ and **(B)** CD4+ T cells stimulated by NECA (1 μM) *in vitro*. **(C)** MFI of pCREB in tumor samples of mice (n=5 in each group).

To determine whether DZD2269 relieves the immunosuppressive effects of adenosine on T cells, we detected pCREB levels in T cells. In *in vitro* assay, DZD2269 inhibited CREB phosphorylation stimulated by 1 μM NECA in CD8+ T cells, with an IC_50_ of approximately 3 nM (Figure 1A). Similar results were observed in CD4+ T cells (Figure 1B, and Supplemental figure S1). In *in vivo* assay, mice were orally administered with DZD2269 (3 or 10 mg/kg, twice a day) for 3 days before peripheral blood was collected. Pre-treatment with DZD2269 blocked CREB phosphorylation (Figure 1C).

### IR induced adenosine release *in vitro* and *in vivo*

**Figure 2:**
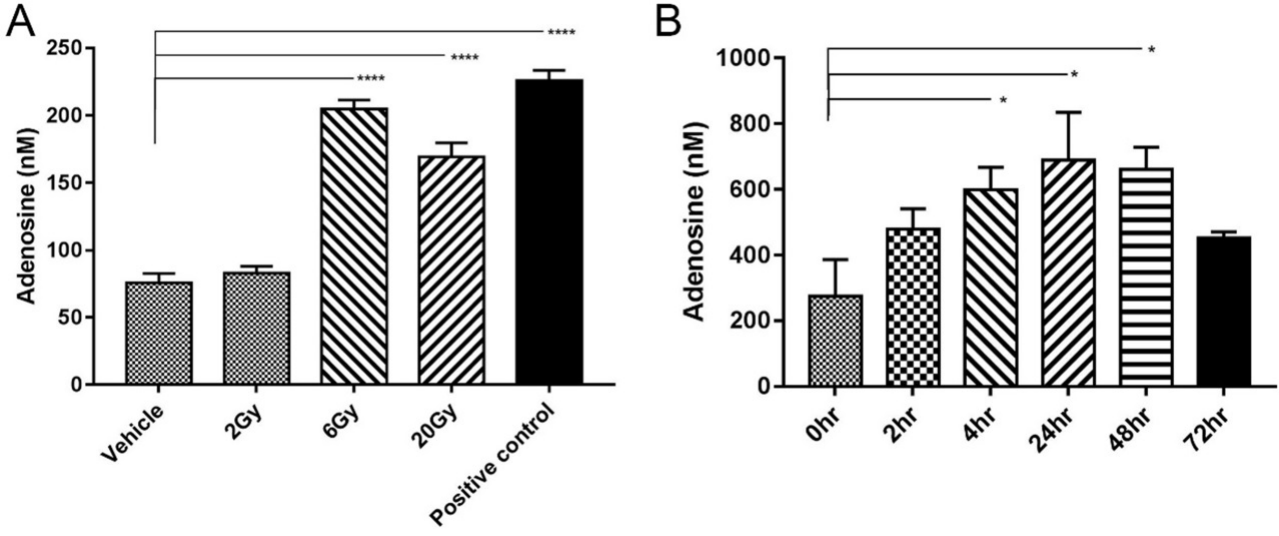
** IR induced adenosine release. (A)** Adenosine concentration in medium (n=3). Samples were collected 24 h after irradiation. **(B)** Free extracellular adenosine concentrations at different time points after a single 5-Gy dose of radiation (n=4 mice in each group). Error bars represent standard error of the mean. Significance was calculated using One-way ANOVA t-test. *p<0.05, **p<0.01, ***p<0.001.

IR-induced tumour cell death may lead to the direct release of adenosine 15. To verify this possibility, MC38 tumor cells were irradiated with different doses of radiation, and the concentration of adenosine in cell supernatant was detected by HPLC. Tumor cells physically disrupted by repeated freezing and thawing were used as positive controls. The adenosine concentration significantly increased after IR at a dose of 6 Gy or 20 Gy but not at a dose of 2 Gy (Figure 2A).

To determine the concentration of adenosine *in vivo*, microdialysis probes were surgically implanted into established MC38 tumor-bearing mice (n=4). IR with a single 5-Gy dose was performed on the tumor areas and samples were collected at 2, 4, 24, 48 and 72 h after IR. Free extracellular adenosine concentrations in the TME were approximately 200-250 nM without IR treatment, similar to the levels in previous report 16. IR caused a rapid increase in adenosine level, which could be observed as early as 2 h. The peak level of approximately 600 nM appeared 48 h later but declined to 400 nM at 72 h (Figure 2B).

### DZD2269 enhanced IR-induced antitumor response

**Figure 3:**
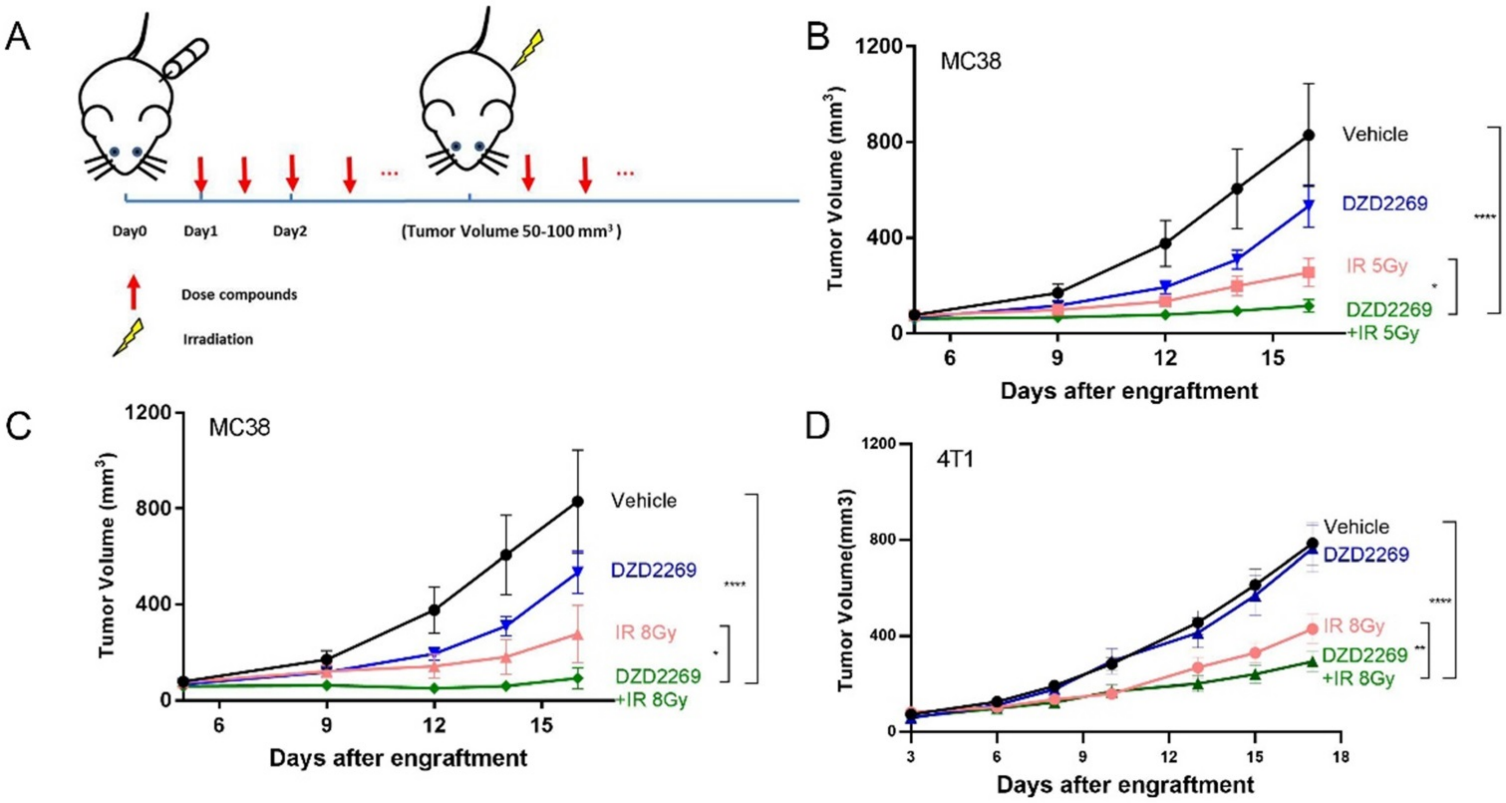
** DZD2269 enhanced IR-induced antitumor response. (A)** Procedure of tumour engraftment and treatment (n=7). **(B-C)** DZD2269 (3 mg/kg) or irradiation monotherapy resulted in incomplete antitumor response in MC38 tumour model (n=9). Combined administration of DZD2269 and irradiation with a single dose of 5 Gy (B) or 8 Gy (C) significantly inhibited tumour growth. **(D)** Similar results were observed in 4T1 tumour model with an irradiation dose of 8 Gy. Significance was calculated using two-way ANOVA. *p<0.05, **p<0.01, ***p<0.001, ****p<0.0001.

Enriched adenosine can mitigate the immune effect of radiotherapy and induce radiation resistance 17. A2AR inhibitor may counteract the immunosuppressive effects of adenosine and enhance the antitumor efficacy of radiation. Therefore, MC38 or 4T1 tumor cells were subcutaneously injected into the back of syngeneic C57BL/6 or Balb/c mice, respectively. The mice were orally administered with DZD2269 (3 mg/kg) or vehicle control twice a day 24 h after engraftment. IR was given to the local tumor area 4-5 days after engraftment when the average tumour volume was 50-100 mm^3^ (Figure 3A). DZD2269 treatment led to incomplete inhibition of tumour volume of approximately 35% in MC38 model (Figure 3B, C), but not in 4T1 model (Figure 3D). Both MC38 and 4T1 models were sensitive to IR, with suppression ratios of 45.2%-69.2%. Compared to either monotherapy, combined treatment with IR and DZD2269 led to synergistic repression of tumor growth (Supplemental figure S2). All treated mice did not display any signs of distress or significant weight loss.

### DZD2269 enhanced T cell infiltration after IR

IR is known to remodel tumor immune microenvironment, such as the upregulation of MHC-I expression and the recruitment of effector T cells 18, 19. To examine the changes in tumor immune microenvironment, MC38 tumor cells were engrafted into female syngeneic C57BL/6 mice, which were then treated with DZD2269 or/and radiation. Tumor-infiltrating lymphocytes expressing CD8, CD4 and Forkhead box 3 (Foxp3) were detected by IHC.

**Figure 4:**
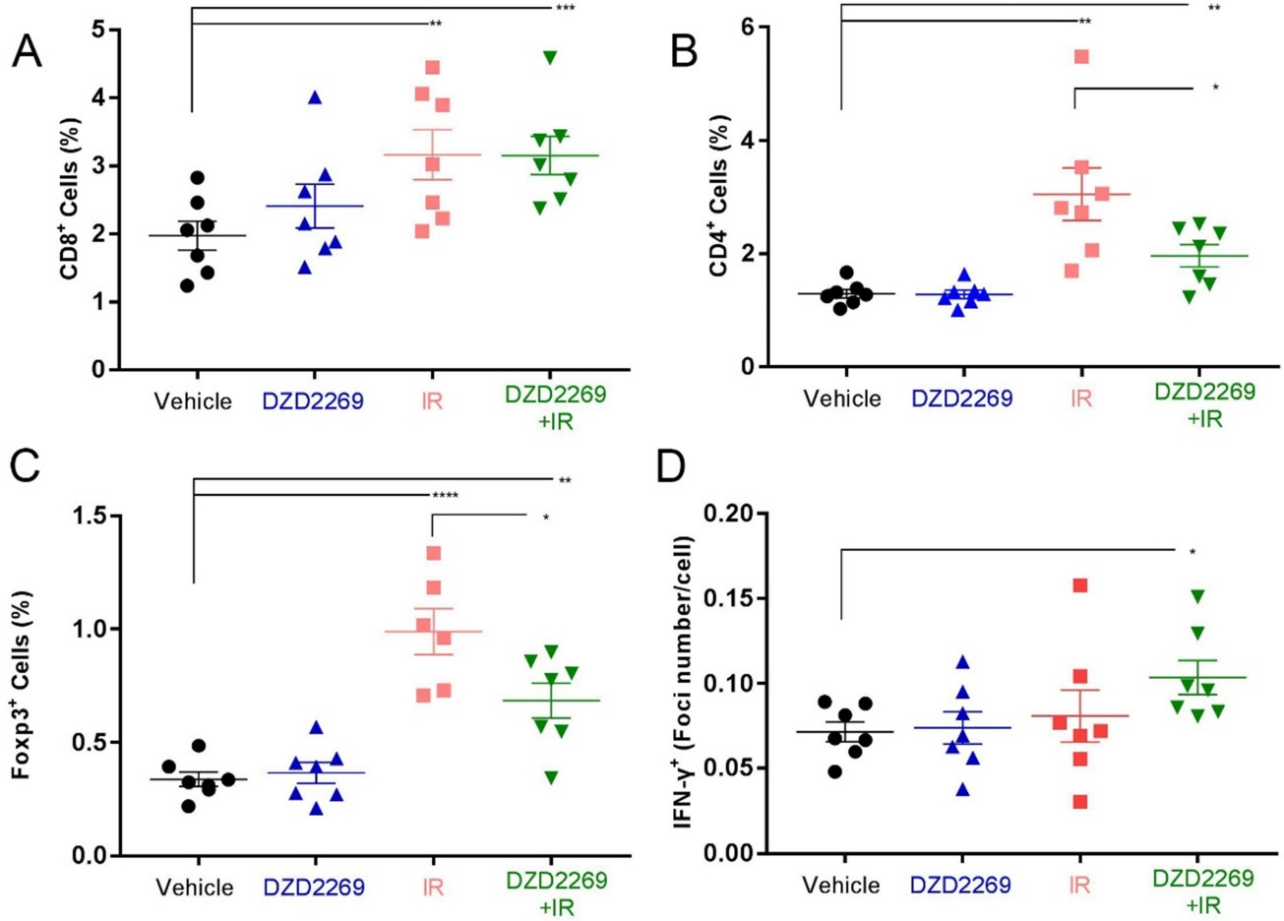
** T cell infiltration and IFN-γ expression in tumor samples. (A)** CD8+ T cell infiltration into MC38 tumors increased following treatment with irradiation, but no further enhancement occurred after combination with DZD2269. **(B-C)** Irradiation induced increased infiltration of CD4+ (B) and Foxp3+ (C) T cells, which could be mitigated by DZD2269. **(D)** IFN-γ expression was increased by DZD2269 in combination with irradiation. Significance was calculated using one-way ANOVA t-test (n=7). *p<0.05, **p<0.01, ***p<0.001.

While IR increased the accumulation of CD8+ lymphocytes, no further enhancement was observed after combination with DZD2269 (Figure 4A). The infiltration of CD4+ lymphocytes increased after IR, but combination treatment with DZD2269 suppressed IR-induced recruitment of CD4+ (Figure 4B). Similarly, combination treatment with DZD2269 suppressed IR-induced recruitment of Treg cells (Figure 4C) (Supplemental figure S3-5).

### IR combined with DZD2269 enhanced IFN-γ expression in tumour microenvironment

Cytokines such as IFN-γ can predict the response to immune checkpoint inhibitors 20. T cell function-related gene expression was reported to be associated with the therapeutic efficacy of A_2A_R blockade 16. To evaluate the correlation between therapy efficacy and T cell function, IFN-γ mRNA expression in tumor tissues was measured by ISH. IR combined with DZD2269 significantly enhanced the mRNA expression of IFN-γ (Figure 4D) (Supplemental figure S6).

## Discussion

Reprogramming of energy metabolism and evading immune destruction are two emerging hallmarks of cancer, providing new insights for cancer therapy 21. With the recent discovery of specific mutations in metabolic enzymes in certain cancer types, metabolic pathway are increasingly considered as source of new targets 22. Meanwhile, treatment of cancer has been revolutionized by immunotherapy with currently widely used checkpoint inhibitors against regulatory immune checkpoint molecules. Adenosine and its receptors are novel targets of checkpoint inhibitors for cancer immunotherapy. Extracellular adenosine is mainly formed by the hydrolysis of tumor cell-derived ATP through the membrane-associated 'tandem ecto-enzymes' CD39/CD73. Adenosine generation in the tumour bulk is mediated by hypoxia-dependent activation of hypoxia-inducible factor 1α. Hypoxia is common in solid tumors and is caused by increased O2 consumption and delayed angiogenesis. Hypoxia causes immunosuppression, promoting tumor recurrence and leading to the failure of radiotherapy 23. The hampered antitumor immunity in hypoxic tumors is mainly mediated by adenosine receptor signalling, especially A_2A_R 24.

Our study confirmed that IR enriched extracellular adenosine, which can thwart the antitumor response 17. Adenosine is widely involved in intracellular metabolic activities 25. Radiation induced cell death releases intracellular adenosine into the extracellular environment, and also releases ATP, which can be hydrolyzed into adenosine via CD39/CD73 pathway 26. On the other hand, radiation can activate DNA repair, which is an energy-consuming process and finally promote ATP consumption and increase adenosine production 22, 27. Therefore, the enriched adenosine level might result from three aspects: i) direct release of intracellular adenosine, ii) activation of DNA repair and iii) hydrolysis of tumor cell-released ATP via the CD39/CD73 pathway.

The immunosuppressive effect of adenosine has been described 17, 28, 29. In addition, A2_A_R plays a crucial role in the attenuation of immune function 30. The accumulated extracellular adenosine in the TME can stimulate A_2A_ and A_2B_ receptors, and inhibit T and natural killer (NK) cell function as well as macrophage activation 31-34. In addition, activation of A_2A_R can promote the differentiation and proliferation of tumor-promoting immune cells, including Treg cells and myeloid-derived suppressor cells (MDSCs) 35. Inhibition of the adenosine signaling pathway impairs the growth and metastasis of solid tumours 13, 36. It has also been reported that adenosine can upregulate vascular endothelial growth factor expression 37. Therefore, adenosine can support tumor angiogenesis.

Immune checkpoint inhibitors (ICIs), such as anti-PD-1/PD-L1 antibodies, have shown good synergistic antitumor effects with IR 38, 39. IR-induced cancer cell death leads to antigen release, which then activates and recruits effector T cells to the tumor area 40, 41. However, IR could cause immunosuppressive effect, with the accumulation of Treg cells as well as the release of immunosuppressive molecules, such as TGF-β 42. Upregulation of immune checkpoint molecule expression in irradiated tumor cells is another major mechanism of IR-induced immunosuppression. Combined IR and immune checkpoint inhibition has produced promising results in clinical trials 38, 39. Our study is the first to demonstrate synergistic antitumor effect produced by IR and A_2A_R inhibitor. Our results proved that A_2A_R antagonist DZD2269 can inhibit CREB phosphorylation induced by adenosine* in vivo* and *in vitro*. We also showed the antitumor effect of DZD2269 on colon and breast cancer in murine models. A_2A_R antagonism can protect immune cells from the immunosuppressive effect caused by pre-existing extracellular adenosine accumulation as well as IR-induced adenosine release in the TME. Further studies are necessary to screen novel compounds of A_2A_R antagonist with better antitumor effects and less side effects from natural products 43,44.

Both effector T cells and Treg cells were recruited 7 days after IR. DZD2269 showed radiosensitive effect that reduced Treg cell infiltration and activated effector T cell function. However, for highly radiosensitive MC38 tumor model, we chose a moderate dose of IR, which was below the reported optimal immune excitation dose (8-12 Gy) 45, The maximum immunostimulatory effect of IR may not have been produced in this situation. DZD2269 may produce a better synergistic antitumor effect with fractionated radiotherapy or stereotactic body radiotherapy (SBRT) than with single-dose radiotherapy in prospective clinical trials.

In summary, our study showed that DZD2269 is a potent and selective A_2A_R antagonist with antitumor effect on murine models. A_2A_R antagonists can be a novel immunotherapeutic strategy in combination with IR to inhibit IR-induced recruitment of Treg cells and activate T cell function through enhancing the expression of IFN-γ. Clinical trials are needed to explore the efficacy and toxicity of combined treatment with IR and A_2A_R antagonist.

## Supplementary Material

Supplementary figures.

## Abbreviations

A_2A_R: adenosine 2A receptors
CREB: cAMP response element-binding protein
IR: irradiation
Treg cells: regulatory T cells
TME: tumour microenvironment
NIH: National Cancer Institutes of Health
ATCC: American Type Culture Collection
5-ITU: 5-iodotubericidin
IACUC: Institutional Animal Care and Use Committee
HPLC: high-performance liquid chromatography
MFI: mean fluorescence intensity
TBST: Tris buffered saline with Tween-20
NECA: 5'-N-ethylcarboxamidoadenosine
pCREB: phosphorylated CREB
NK cells: natural killer cells
MDSCs: myeloid-derived suppressor cells
VEGF: vascular endothelial growth factor
SBRT: stereotactic body radiotherapy
