# Supplementary material for: A2AR Antagonism with DZD2269 Augments Antitumor Efficacy of Irradiation in Murine Model
Source: J Cancer. 2020 Mar 26;11(12):3685–92. doi: 10.7150/jca.43966 (PMC7150468; doi:10.7150/jca.43966)
Supplement: Supplementary file 1 — Supplementary figures. [file jcav11p3685s1.pdf]

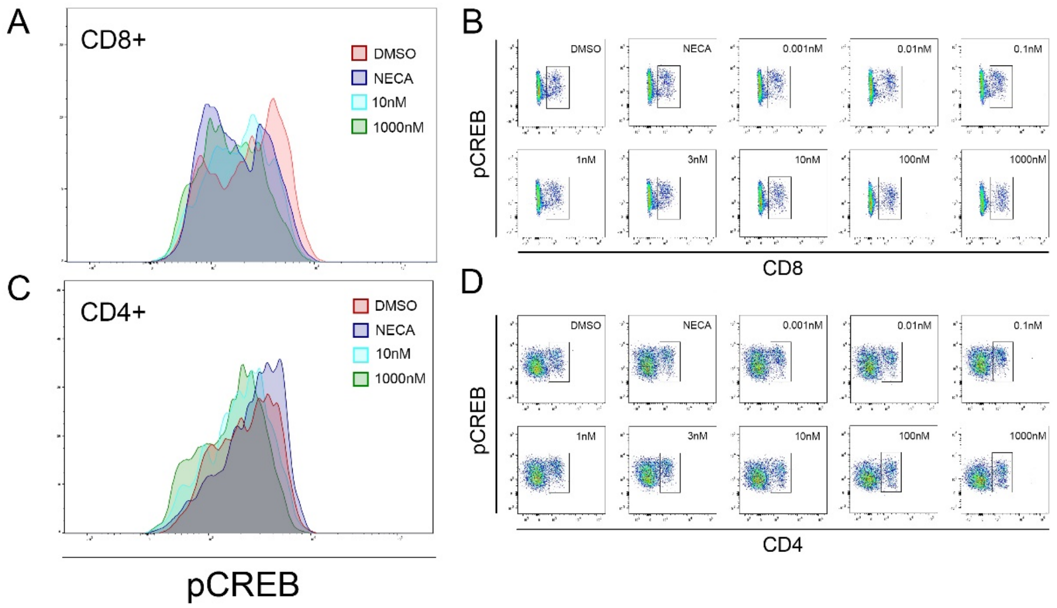

Figure S1. Blockage of A2AR with DZD2269 inhibited CREB phosphorylation in mouse blood cells. (A-D) Flow cytometry histogram/plots images.

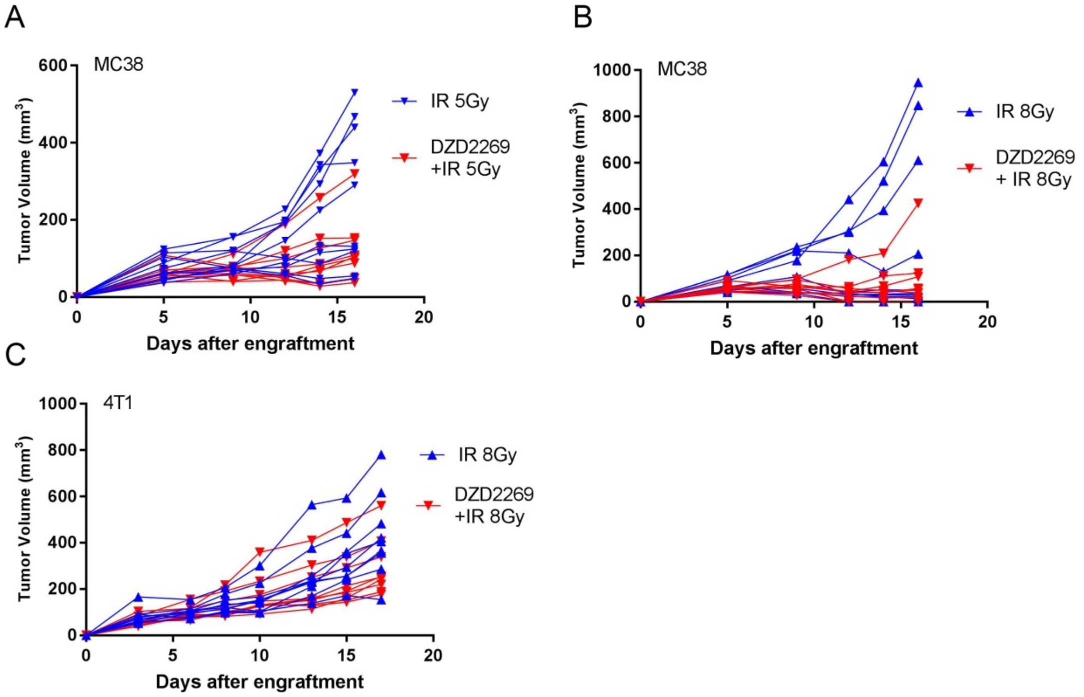

Figure S2. Spider plots of individual MC38 (A-B) and 4T1 (C) tumor bearing mice treated with irradiation only or combination therapy with DZD2269 (3 mg/kg).

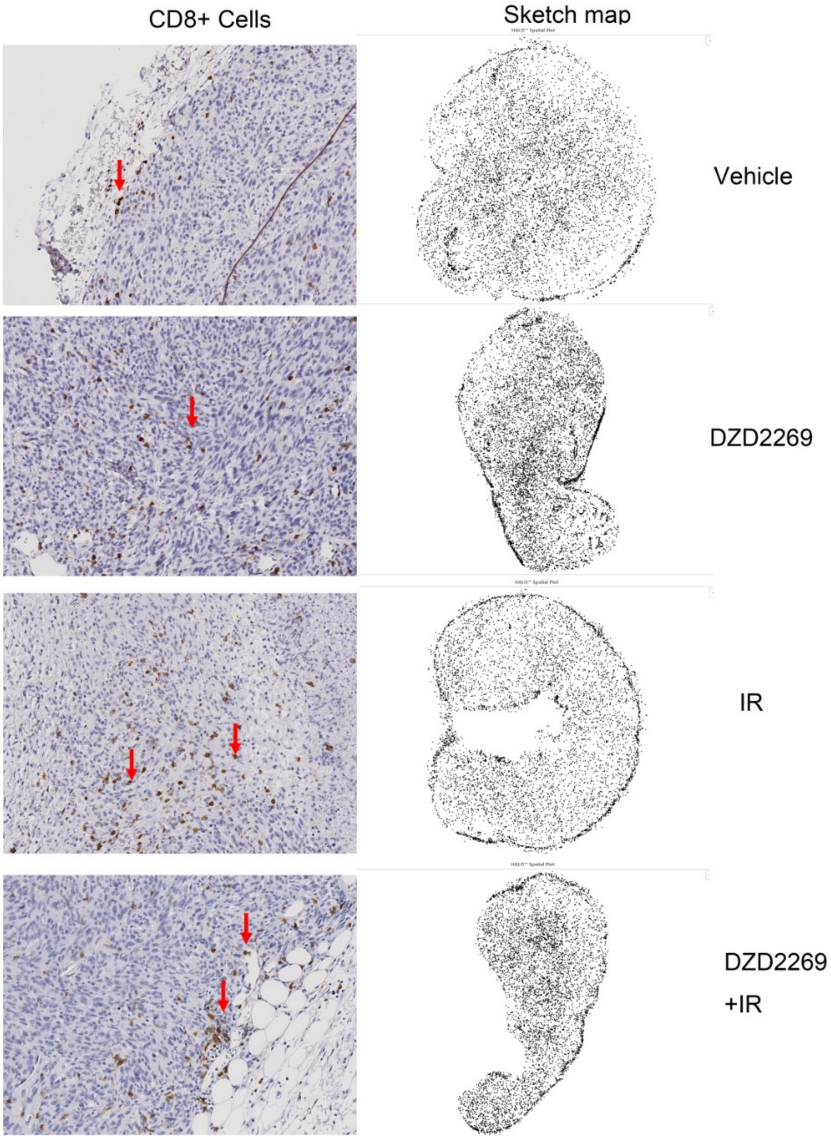
CD8+ Cells
Sketch map
Vehicle
DZD2269
IR
DZD2269
+IR

Figure S3. Immunohistochemistry (20×) and sketch map of CD8+ tumor infiltrating lymphocyte of MC38 tumor after combined treatment of DZD2269 and IR.

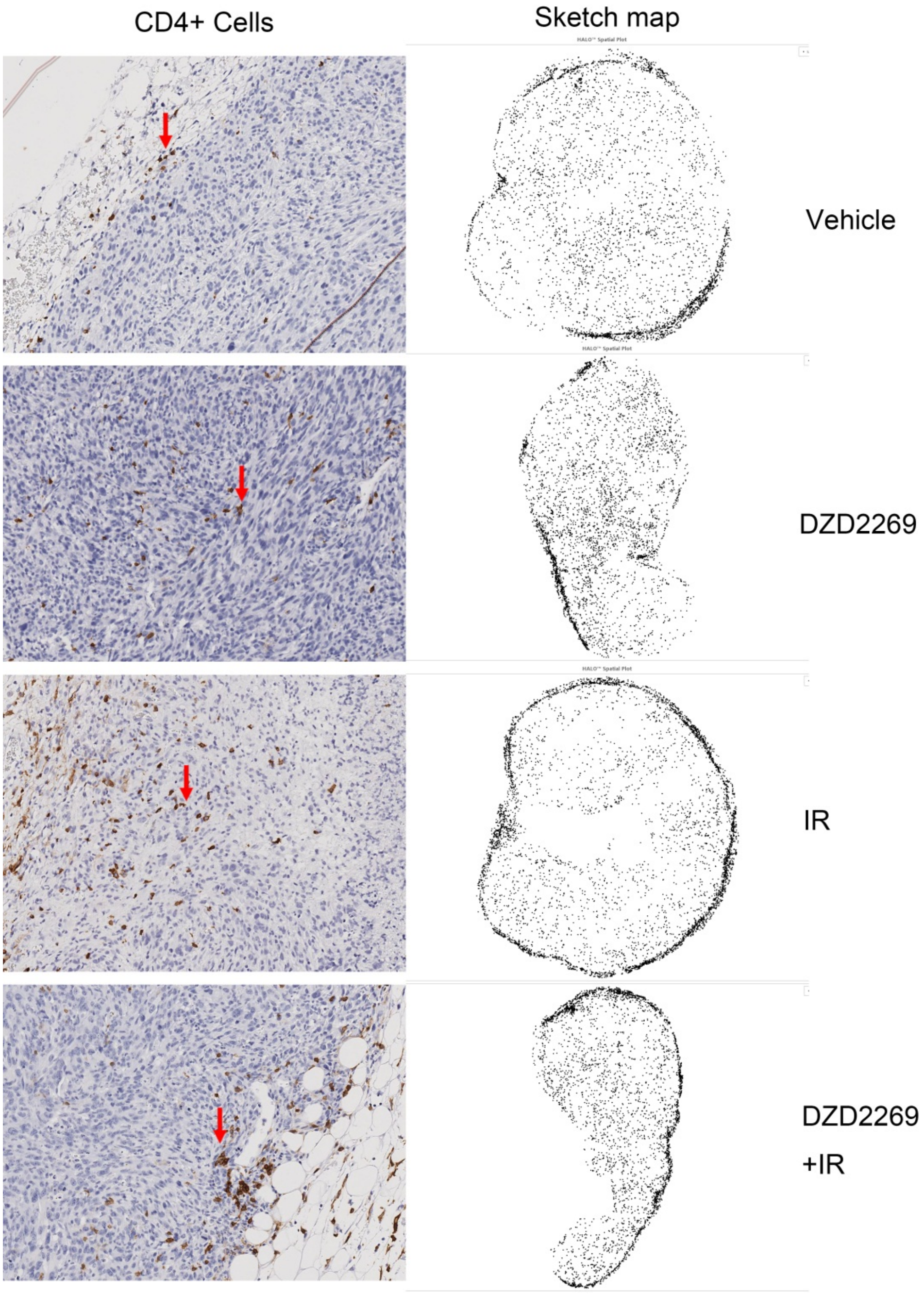

Figure S4. Immunohistochemistry (20×) and sketch map of CD4+ tumor infiltrating lymphocyte of MC38 tumor after combined treatment of DZD2269 and IR.

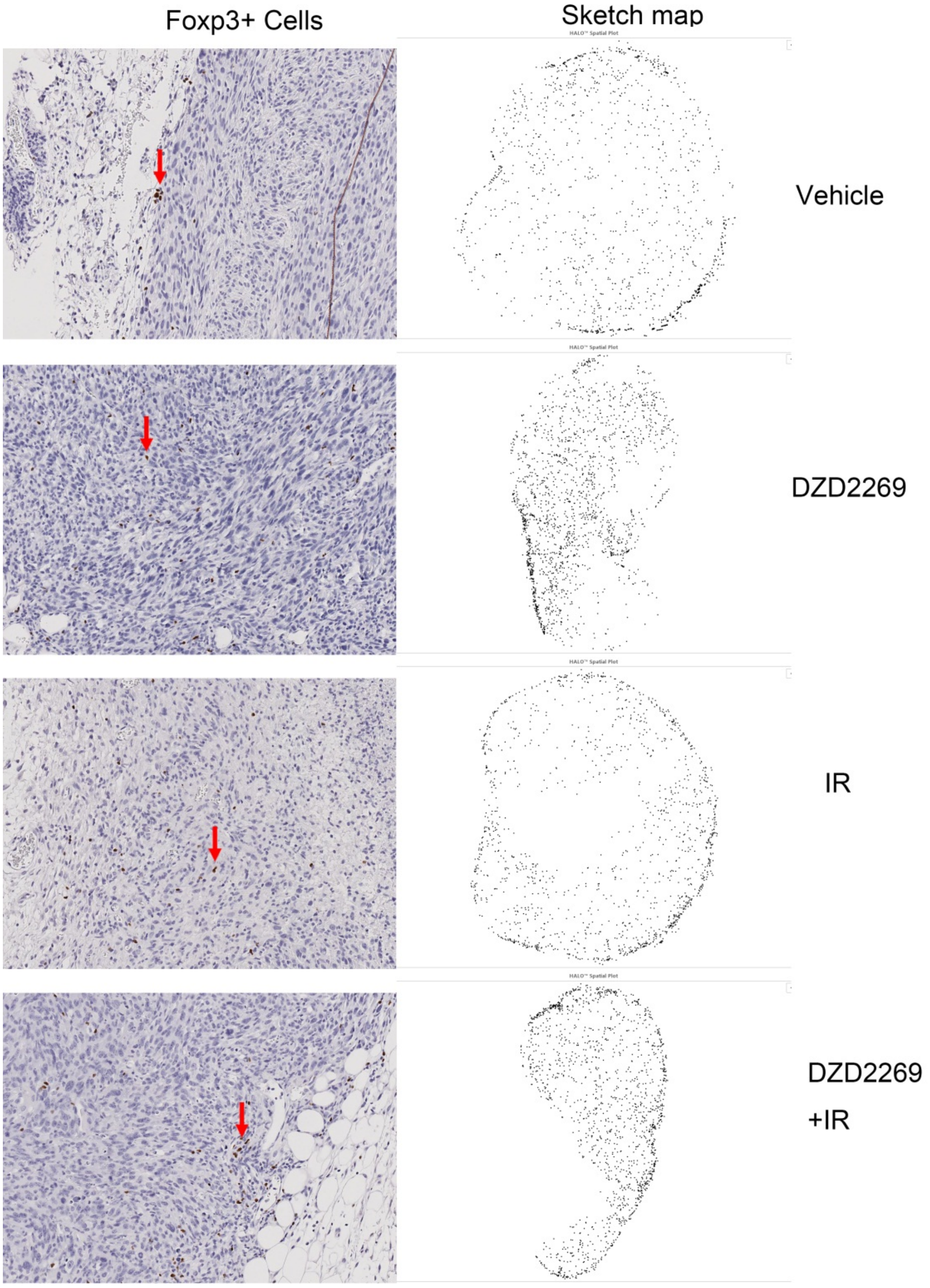

Figure S5. Immunohistochemistry (20×) and sketch map of Foxp3+ tumor infiltrating lymphocyte of MC38 tumor after combined treatment of DZD2269 and IR.

IFN-γ

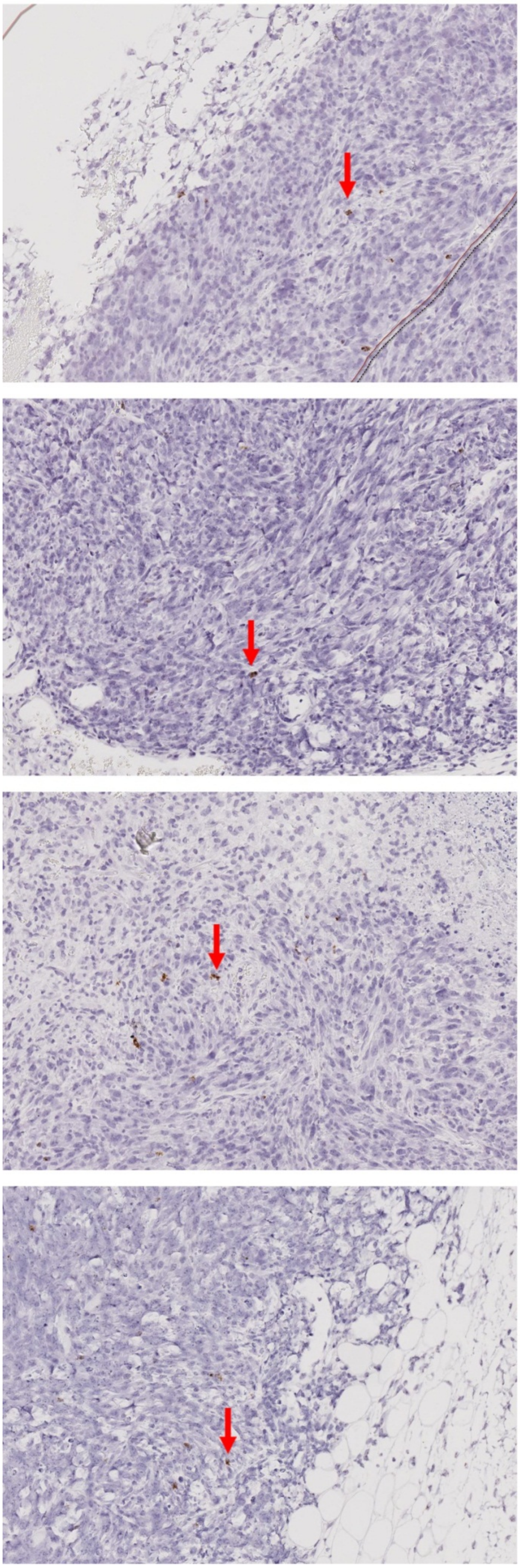

Vehicle

DZD2269

IR

DZD2269
+IR

Figure S6. ISH analysis of IFN-γ expression (20×) within tumor infiltrating lymphocyte of MC38 tumor after combined treatment of DZD2269 and IR.
